# Fistula Lachrymalis Caused by Syphilis

**Published:** 1851-04

**Authors:** 


### ARTICLE XV.

### *Fistula Lachrymalis caused by Syphilis.*

Dr. Tavignot sketches, in the *Journal des Connaissances Medico-Chirurgicales*, a rapid history of this form of fistula; it is the tertiary form of syphilis, which occasions the disease of the lachrymal sac; the ascending branch of the superior maxillary having been invaded by osteitis or periostitis. The diagnosis reposes upon three orders of signs: 1. The succession of previous symptoms, the appreciation of which is not always an easy matter, particularly if mercury, having been exhibited, has interfered with the regular succession of morbid manifestations; the recollections of the patient, and the various stains which the disease may have left upon the body, will, however, in most cases, powerfully assist the diagnosis. 2. The local symptoms of the fistula; and, 3. The concomitant syphilitic manifestations. When the latter are present, the task of the surgeon is greatly simplified; but in some cases it is not so, and, the fistula lachrymalis being the only symptom observable, it becomes necessary to examine if, under the influence of syphilis, it may not present some characteristic local appearances. In simple fistula, when the contents of the sac have been removed, the bony structure of the diseased region is found in a state of perfect regularity—a fact which is easily ascertained by comparison with the healthy side. When syphilis has produced the deformity, on the contrary, in most cases a hard swelling may be felt on the side of the nose, in the region corresponding to the nasal duct. The os unguis, the nasal bones, or the angular prominence of the os frontis, may also be affected with osteitis, and betray the nature of the case by their tumefaction. Often, in these cases, the superior maxillary being also diseased, the incisor teeth are movable, not *in* their alveoli, but *with* them, showing that a portion of the bone is loosened. The treatment recommended is, in the first place, that which experience has proved to be most efficient in tertiary syphilis, namely, hydroiodate of potass, in doses of from ten grains to one drachm daily. When the swelling of the bones has subsided, if the symptoms of fistula lachrymalis have not disappeared, the usual treatment of this affection should be resorted to, the specific character of the malady having been removed, its consequences still persisting.

